# Reduced developmental competence of immature, in-vitro matured and postovulatory aged mouse oocytes following IVF and ICSI

**DOI:** 10.1186/1477-7827-6-58

**Published:** 2008-12-01

**Authors:** Orly Lacham-Kaplan, Alan Trounson

**Affiliations:** 1 Monash Immunology and Stem Cell Laboratories, Monash University, Clayton, Victoria, Australia; 2 California Institute for Regenerative Medicine, San Francisco, CA, USA

## Abstract

**Background:**

The present study highlights basic physiological differences associated with oocyte maturation and ageing. The study explores the fertilizing capacity and resistance to injury of mouse oocytes at different stages of maturation and ageing following IVF and ICSI. Also, the study examines the developmental competence of embryos obtained from these oocytes. The outcome of the study supports views that the mouse can be a model for human IVF suggesting that utilizing in-vitro matured and failed fertilized oocytes to produce embryos mainly when limited number of oocytes is retrieved in a specific cycle, should be carefully considered.

**Methods:**

Hybrid strain mouse oocytes were inseminated by in-vitro fertilization (IVF) or intracytoplasmic sperm injection (ICSI). Oocytes groups that were used were germinal vesicle (GV) in-vitro matured metaphase II (IVM-MII), freshly ovulated MII (OV-MII), 13 hrs in-vitro aged MII (13 hrs-MII) and 24 hrs in-vitro aged MII (24 hrs-MII). Fertilization and embryo development to the blastocyst stage were monitored up to 5 days in culture for IVF and ICSI zygotes. Sperm head decondensation and pronuclear formation were examined up to 9 hrs in oocytes following ICSI. Apoptotic events in blocked embryos were examined using the TUNNEL assay. Differences between females for the number and quality of GV and OV-MII oocytes were examined by ANOVA analyses. Differences in survival after ICSI, fertilization by IVF and ICSI and embryo development were analysed by Chi-square test with Yates correction.

**Results:**

No differences in number and quality of oocytes were identified between females. The findings suggest that inability of GV oocytes to participate in fertilization and embryo development initiates primarily from their inability to support initial post fertilization events such as sperm decondensation and pronuclei formation. These events occur in all MII oocytes in similar rates (87–98% for IVF and ICSI). Following ICSI, pronuclei appeared in IVM and freshly ovulated oocytes by 8–9 hrs after insemination. In comparison, pronuclei appeared in 13 hrs aged oocytes by 4–5 hrs. Significantly higher proportions (P < 0.001) of blastocysts resulted from OV-MII oocytes than the other groups examined with 75% and 71% for IVF and ICSI, respectively. The 13 hrs-MII oocytes resulted in 47 and 40% blastocysts, while IVM-MII and 24 hrs-MII oocytes resulted in 38% and 0% blastocysts from IVF and 5% and 5% from ICSI, respectively. In addition, anucleate cells and DNA fragments were observed in retarded embryos derived from IVM and aged oocytes, however, apoptotic events were similar for all groups.

**Conclusion:**

The data suggests that the use of oocytes other than freshly ovulated MII should be carefully considered for assisted reproduction.

## Background

Mammalian oocyte develops into a viable embryo following fertilization, when sperm penetration takes place within 4–6 hrs from ovulation. After this time, the capacity of the oocyte to fertilize and to develop normally is dramatically reduced [1,2]. Even though there are many compiling evidence that freshly aspirated metaphase II oocytes are the most optimal in producing viable embryos, some IVF clinics around the world still utilize in-vitro matured and failed fertilized oocytes to obtain embryos.

Germinal vesicle (GV) stage oocytes have no ability to participate in the early steps of fertilization. Although sperm penetration has been reported in GV oocytes [3], sperm head remodelling does not progress beyond slight swelling. In many species, an increase in sperm remodelling capability is acquired following a period of in-vitro maturation [4-8]. It has been noted, however, that factors such as follicle size, hormonal priming and the presence of follicle stimulating hormone (FSH) in the culture medium affect the rate of preimplantation development of IVM oocytes [4,9]. Even so, developmental competence of oocytes matured in-vitro is compromised in most species investigated.

Problems in embryo development are also expected when oocytes are inseminated after extend culture time. In mice, oocytes that are inseminated or injected with sperm 24 hrs or longer after retrieval from the oviducts have abnormal calcium oscillation patterns leading to increased apoptosis and retarded embryo development [10,11]. These events accelerate if oocytes are cumulus intact [12] and are similar to events occurring when in-vivo aged mouse oocytes are retrieved from the oviducts at 18.5 hrs after ovulation [13].

The present study examines differences between immature, in-vitro matured, freshly ovulated and in-vitro aged oocytes following in-vitro fertilization (IVF) and intracytoplasmic sperm injection (ICSI). The influence that maturation stage and ageing of mouse oocytes has on fertilization, resistance to injection, pronuclei formation and embryo developmental competence to the blastocyst stage was investigated. The study utilizes hybrid F1 mouse strain, which has no developmental restrictions in culture following in-vitro insemination, allowing the use of simple salt solutions as the culture medium. To avoid bias, eggs were retrieved from same age females receiving similar hormonal treatments. Oocytes were retrieved from females and exposed to same culture conditions and treatments. The survival rate of the different oocytes to ICSI was recorded and sperm head swelling and pronuclear formation in intact oocytes were examined at different times after ICSI. The capacity of the resultant zygotes to develop to blastocysts on day 5 of culture and the chromatin integrity and apoptotic events of retarded and normal embryos on that day were examined by immunofluorescence.

## Methods

### Research plan

Female mice at 6 weeks of age were hormonally treated. After the first hormonal injection, some females were used to retrieve germinal vesicle (GV) oocytes obtained from the ovaries. These oocytes were used either for IVF and ICSI or cultured in-vitro for 13 hrs for spontaneous maturation. This group is the in-vitro matured (IVM) oocyte group. The rest of the females were subjected to a second hormonal injection to induce ovulation. These females were used to obtain freshly ovulated oocytes. Freshly ovulated oocytes were either used for IVF and ICSI or left in-culture to age for either 13 hrs or 24 hrs. Aged oocytes were also used for IVF and ICSI. The above plan resulted in five oocyte groups: GV, IVM-MII, OV-MII, 13 hrs-MII and 24 hrs-MII.

### Rational of the study

Total of 5 groups were included in the study attempting to use similar groups that are used in IVF clinics to produce embryos: 1) GV oocytes; 2) IVM oocytes (matured for 13 hrs in vitro); 3) Freshly ovulated MII oocytes; 4) 13 hrs in-vitro aged oocytes; 5) 24 hrs in-vitro aged oocytes. These groups represent pre mature, mature MII oocytes obtained in-vivo and in-vitro, and in-vitro aged. Group 3 the freshly ovulated MII oocytes is the control group. The outcome of fertilization and embryo development of the other groups was compared to this group.

### Culture media

Solutions used in the study were prepared from analytical grade chemicals (BDH Chemicals Pty., Ltd., Melbourne, Australia; Sigma Chemicals Co., St. Louis. MO, US). Sperm were collected and capacitated in modified Tyrode's medium [14]. Oocytes and embryos were handled in M2 medium [15] and cultured in M16 medium [16].

### Animals

Hybrid F1 (C57BLxCBA) mice were used for the experiments. Animal protocols were approved by the Monash University Animal Ethics Committee (SOBS-A) and conducted under the Australian National Health and Medical Research Council (NHMRC) code of practice.

### Preparation of gametes

#### Oocytes

Six-week old females were given a single subcutaneous injection of 10 IU Pregnant Mare's Serum Gonadotrophin (PMSG, Folligon, Intervet, Lane Cove, Australia). To obtain GV oocytes, females were killed 48 hrs after PMSG injection and the dissected ovaries placed in 2 ml of M2 handling medium. Antral follicles were pierced with sharp fine forceps to release the oocytes. Cumulus cells were mechanically removed. Denuded GV oocytes were transferred into 20 μl drops of M16 culture medium until they were used for injection or insemination. For spontaneous IVM, cumulus intact and cumulus free GV oocytes were collected and cultured in M16 medium for 12–13 hrs. At that time oocytes were exposed to 100 IU/ml hyaluronidase in M2 medium (Type IV-S, Sigma Chemicals Co., St. Luis, Mo., USA) for 5 min to allow cumulus cells to separate from the oocytes. Oocytes that extruded the first polar body were considered to have matured to MII stage. These IVM-MII oocytes were washed in M2 free of hyaluronidase and were subjected to IVF and ICSI. To obtain OV-MII oocytes, females were injected subcutaneously with human Chorionic Gonadotrophin (hCG, Chorulon, Intervet) 48 hrs after the PMSG injection. At 12–13 hrs after hCG injection the females were killed and the cumulus oocyte complexes were released from the oviducts into M2 medium containing to100 IU/ml hyaluronidase to allow cumulus cells to separate from the oocytes. Oocytes washed of adhering cumulus cells were injected or inseminated with sperm. To obtain aged oocytes, cumulus free OV-MII were cultured in M16 for either 12–13 hrs or 23–24 hrs, before IVF and ICSI.

#### Sperm

Sperm were obtained from the cauda epididymes of 8 to 10 week old F1 males. One cauda was dissected out from the body and transferred into previously equilibrated (37°C at 5% CO2 in air) 2 ml MT6 medium under a thin layer of mineral oil (Sigma). Sperm were passively released into the culture medium through a single slit in the base of the cauda. Sperm were capacitated for 2 hrs before IVF and ICSI.

### In vitro fertilization (IVF), intracytoplasmic sperm injection (ICSI) and embryo development

For IVF, oocytes were placed in sperm suspension for 3 hrs and then washed and cultured in M16 medium for further analyses. For ICSI, oocytes were injected using a Piezo injection system (PiezoDrill, Burleigh Instrument Inc., Burleigh Park, NY, USA) mounted on a Leica micromanipulator (Leica Microscopy Systems Ltd, Wetzler, Germany) adjacent to a Leica inverted microscope. Sperm were selected randomly and the sperm heads were separated from the tails using a high Piezo pulse before they were injected singly into each oocyte [17]. Intact oocytes were cultured in 20 μl drops of M16 medium under oil at 37°C in 5% CO2 in air. The number of IVF and ICSI oocytes that had extruded a second polar body and had two pronuclei visible 9 hrs after insemination or injection was recorded. Oocytes with no second polar body and no pronuclei were considered not fertilized. The development of zygotes to blastocysts on day 5 of culture was recorded.

### Formation of pronuclei

In a separate experiment, oocytes at 1–2, 4–5 and 8–9 hrs after ICSI (n = 15 for each oocyte age and time groups) were placed on 70% ethanol washed glass slides and allowed to air dry at room temperature for 3–5 min before they were immersed in 100% methanol repeatedly. Oocytes were stained with 4'6-Diamidino-2-phenyiodole dilactate (DAPI; Roche Applied Science, Castle Hill, Australia) dissolved (1:1000) in anti fade Vectashield mounting medium (Vector laboratories Inc., Burlingame, USA). The slides were examined at absorption and emission wave lengths of 344 nm and 450 nm, respectively, using an Olympus 1 × 70 inverted fluorescent microscope (Olympus Optical Co., Tokyo, Japan).

### Analyses of chromatin integrity and apoptosis

Embryos that were blocked in cleavage and morphologically normal blastocysts on day 5 after IVF or ICSI were fixed on a glass slides using 4% paraformaldehide in pH 7.4 PBS (w/v). Oocytes were examined by the TUNEL assay using the ApopTag plus Fluorescein In Situ Apoptosis Detection Kit (Chemicon International Inc., Temecula, CA) as per manufacturer instructions. Embryos were counterstained with DAPI. A positive apoptosis event was recorded when DNA stained with DAPI was found also to be positive for dUDP nick identified by the fluorescent marker FITC.

### Statistical analyses

Differences between females used for the experiments in relation to ovulation and quality of oocytes were analysed by ANOVA. Survival, fertilization and embryo development data were analysed by Chi-square (χ2) using Yates' correction for samples less than 50. Chi-square test was used to compare the total number between two groups or among several experimental groups. P values of 0.05 or less were considered significant.

## Results

### Fertilization and embryo development

Hormonal treatment resulted in an average of 20 oocytes from each female used with no differences identified between females. A total of 151, 115, 151, 173, 214 oocytes at the GV, IVM-MII, OV-MII, 13 hrs-MII and 24 hrs-MII stages were inseminated with sperm, respectively. Only one GV oocyte showed signs of fertilization 9 hrs after insemination. It was difficult to identify clear signs of fertilization in the 24 hrs-MII oocytes as most underwent spontaneous cleavage or fragmentation within 1–2 hrs from insemination. Fertilization rates for IVM-MII, OV-MII and 13 hrs-MII oocytes were 82%, 94% and 98%, respectively (Table 1). The fertilization rate in the IVM-MII group was significantly lower than ovulated and 13 hrs-MII oocytes (P < 0.001). None of the GV or 24 hrs-MII oocytes developed to the blastocyst stage in culture. The blastocyst rate was significantly higher in the OV-MII group of oocytes (P < 0.001) when compared to rates of IVM-MII and 13 hrs-MII aged oocytes (Table 1).

**Table 1:** Rates of pronuclei formation and development to morula and blastocysts of IVM, MII and aged oocytes following IVF.

	IVM	Ovulated	13 hr Aged
No. oocytes inseminated	115	151	173
No. oocytes with 2 PN/inseminated	94 (82%)^a, b^	142 (94%)^a^	169 (98%)^b^
2 cell embryos/inseminated	89 (77%)^c^	135 (89%)^c, a^	129 (75%)^a^
4 cell embryos/inseminated	61 (53%)^a^	126 (83%)^a, b^	98 (57%)^b^
8 cell embryos/inseminated	55 (48%)^a^	119 (79%)^a, b^	83 (48%)^b^
No. blastocysts/inseminated	44 (38%)^a^	114 (75%)^a, b^	82 (47%)^b^

Similar letters indicate statistical differences between groups of the same developmental stage

^a, b^p < 0.001; ^c^p < 0.01

Totals of 173, 195, 236, 251 and 230 oocytes at the GV, IVM-MII, OV-MII, 13 hrs-MII and 24 hrs-MII aged stages were injected with a single spermatozoon. The survival rate of oocytes following injection increased gradually with maturation and ageing, and was the highest for 24 hrs-MII oocytes (80%). Except for GV oocytes, over 90% of the IVM-MII, OV-MII and 13 hrs-MII oocytes were fertilized. Similar to IVF, after ICSI it was difficult to identify fertilization in 24 hrs-MII oocytes as most cleaved spontaneously within 1 to 2 hrs from injection. IVM-MII, OV-MII and 13 hrs-MII oocytes cleaved to 2-cells at rates of 79% (n = 72), 89% (n = 133) and 90% (n = 139), respectively (Table 2). OV-MII oocytes had significantly higher cleavage rate than IVM-MII (38%; P < 0.05) and 13 hr-MII (47%; P < 0.05) oocytes. The development to blastocysts following ICSI was also significantly higher (P < 0.001) for OV-MII oocytes (71%, n = 106) than the other three groups (5%, n = 5; 40%, n = 62 and 5%, n = 10 for IVM-MII, 13 hrs-MII and 24 hrs-MII aged oocytes, respectively). GV oocytes did not form pronuclei or cleave in culture following ICSI.

**Table 2:** Rates of survival, pronuclei formation and development to morulae and blastocysts of IVM, MII and aged oocytes following ICSI.

	IVM	Ovulated	13 hr Aged
No oocytes injected	195	263	251
No. oocytes survived	91 (47%)^a, b, d^	149 (57%)^a, d^	155 (62%)^a, b^
2 PN	89 (98%)	141 (95%)	145 (94%)
2-cells/survived	72 (79%)^a, c^	133 (89%)^a^	139 (90%)^c^
4-cells/survived	34 (37%)^c^	122 (82%)^c^	107 (69%)^c^
8-cells/survived	16 (18%)^a^	116 (78%)^a^	81 (52%)^a^
No. blastocysts/survived	5 (5%)^a^	106 (71%)^a^	62 (40%)^a^

Similar letters indicate statistical differences between groups of the same developmental stage

^a^p < 0.001; ^b^p,0.005; ^c^p < 0.01; ^d^p,0.05

**Figure 1:**
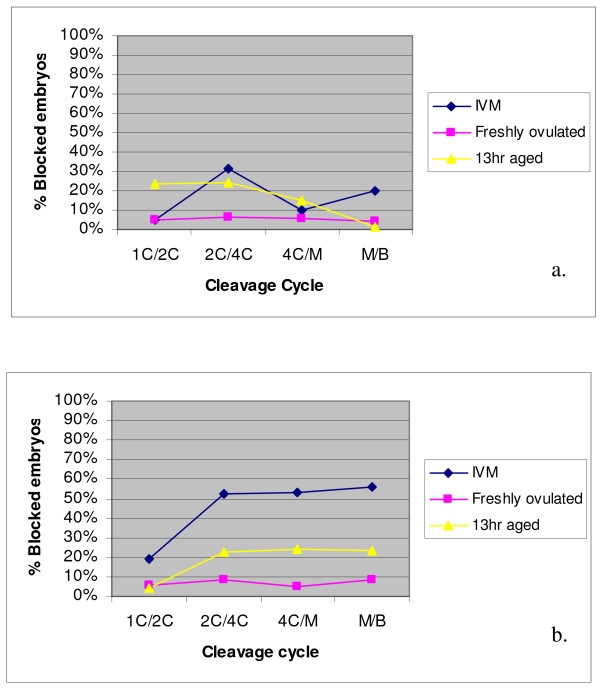
Blockage rate of embryos following IVF (a) and ICSI (b) of embryos obtained from IVM, freshly ovulated and 13 hr aged oocytes.

As presented in figure 1a and 1b a low and constant number of embryos derived from OV-MII oocytes stopped cleaving at each stage of development in-vitro. IVM-MII oocytes derived embryos had the highest proportions of embryos that stoped at the 2-cell and morula/8 cell stages following IVF (30% and 20%) and ICSI (50% and 70%). The proportions of IVF embryos derived from 13 hrs-MII oocytes that blocked in culture was gradually reduced through development. The number of embryos in this group that blocked at the second cell cycle (from 2-cells to 4-cells) was the highest when ICSI was used. The number of embryos that blocked stayed relatively constant after that point.

### Pronuclei formation

**Figure 2:**
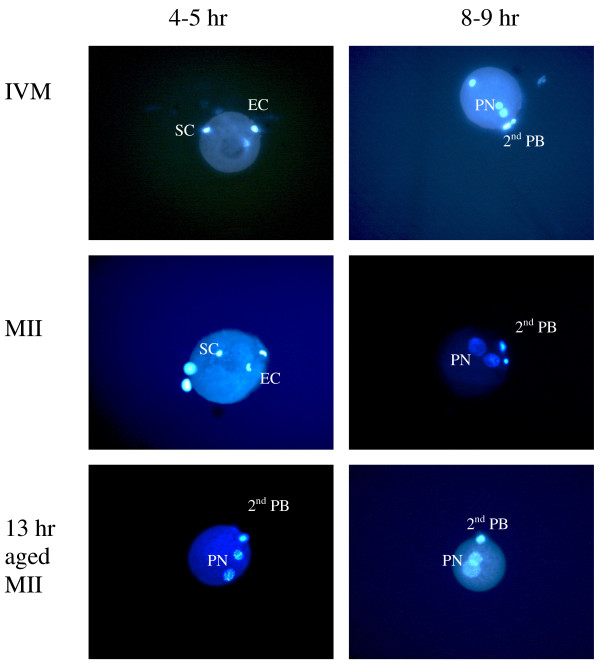
**Pronuclei formation at 4–5 and 8–9 hrs after ICSI of IVM, freshly ovulated and 13 hrs aged oocytes injected at different maturation stages.** Oocytes were injected with a single sperm head. Oocytes were fixed and stained with DAPI at different times after injection. SC = sperm chromatin; EC = egg chromatin; PN = pronuclei; PB = polar body.

A total of 10 oocytes from each of the experimental groups were successfully analysed at 1–2, 4–5 and 8–9 hrs after ICSI and DAPI staining. Sperm heads did not show any changes within 1–2 hrs of ICSI in any of the groups. By 4–5 hrs, most of sperm heads were visibly decondensed in IVM-MII, OV-MII and 13 hrs-MII oocytes (Figure 2). In most (8/10) 13 hrs-MII oocytes, pronuclei were already visible by that time. At 8–9 hrs from injection, fully formed male and female pronuclei were observed in 9/10 of IVM-MII oocytes, 10/10 OV-MII and 9/10 13 hrs-MII oocytes (Figure 2). In all GV oocytes, sperm heads were slightly swollen by 8–9 hrs with no change observed after this. In these oocytes, the female chromatin remained defuse (GVBD) and did not mature to metaphase. In 24 hrs-MII oocytes, pseudo-pronuclei appeared in 4/10 oocytes within 1–2 hrs after ICSI, while sperm heads were still in a condensed form. Sperm heads were swollen by 4–5 hrs in 5/10 oocytes while female pronuclei were already visible. Similar observations were recorded for 24 hrs-MII oocytes 8–9 hrs after ICSI. In this group only 2 oocytes had a second polar body and 2 clear pronuclei, presumably a male and a female, while the rest had one large pronucleus and either swollen sperm head or several DNA patches within the cytoplasm. The second polar body was not extruded from these oocytes leading to poly-nucleated cytoplasm.

### Chromatin integrity and apoptosis

**Figure 3:**
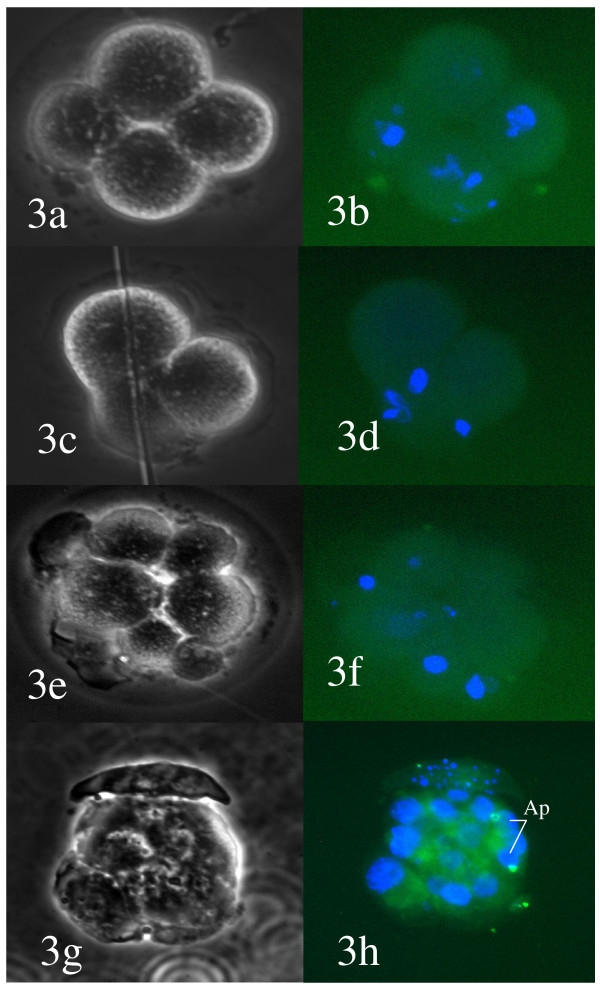
**Blocked embryos derived from 24 hrs in-vitro aged oocytes after ICSI.** Scattered DNA is observed in all embryos. Embryos 3a, 3c, and 3e are not associated with positive TUNEL assay (3b, 3d, 3f). Most embryos contain cell fragments, empty of chromatin. Embryo 3 g has some cells with clear apoptotic (Ap) events identified by the TUNEL assay (3h). Left panel: bright field. Right field: fluorescence images for DAPI and FITC to detect DNA and positive TUNEL.

**Figure 4:**
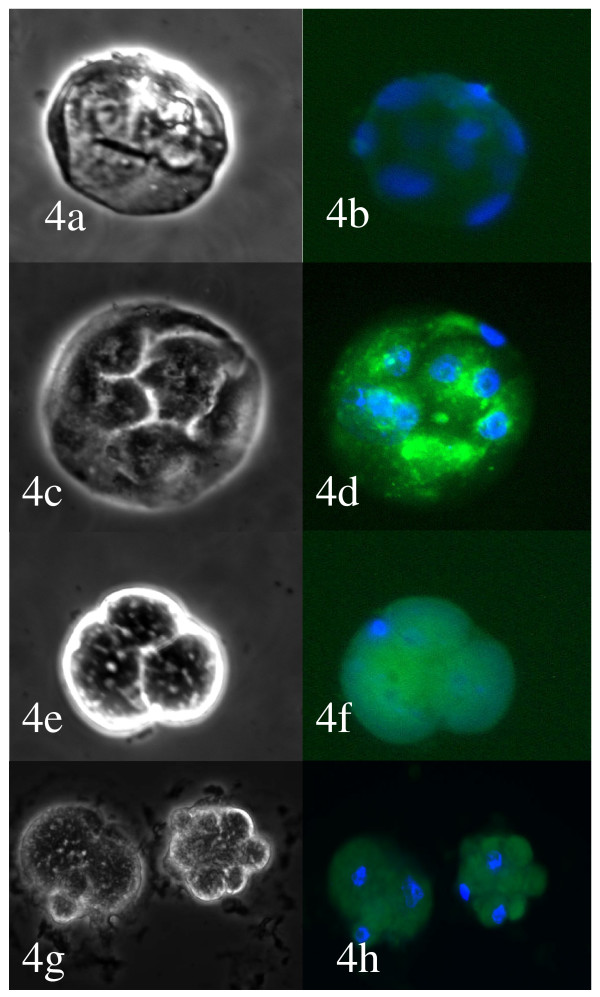
**Blocked embryos derived from IVM oocytes after ICSI.** Scattered DNA and DNA breakages is not obvious. All embryos have blastomeres that are empty of chromatin. Left panel: bright field. Right field: fluorescence images for DAPI and FITC to detect DNA and positive TUNEL.

**Figure 5:**
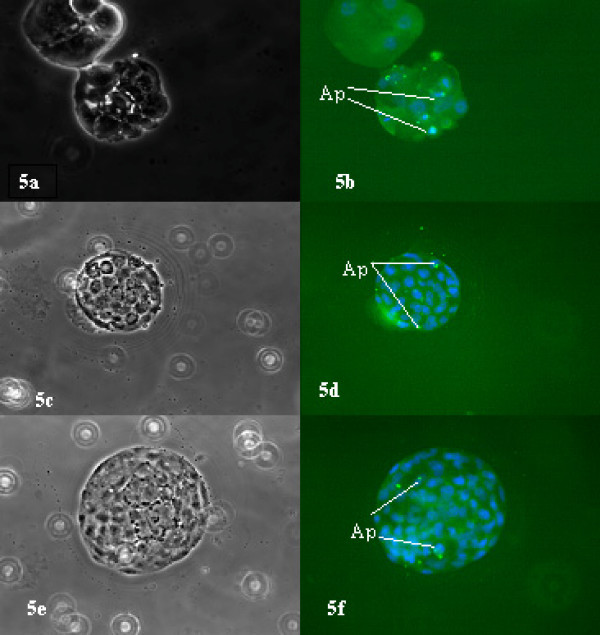
**Blastocyst stage embryos developed from IVM oocytes (5a, b), 13 hrs aged oocytes (5c, d) and from freshly ovulated oocytes after ICSI (5e, f).** Some cells within blastocysts in all groups were positive (Apo) to the TUNEL assay indicating apoptotic events. Left panel: bright field. Right field: fluorescence images for DAPI and FITC to detect DNA and positive TUNEL.

Blocked embryos and embryos at the morula and blastocyst stages after IVF and ICSI of IVM-MII, OV-MII,13 hrs-MII and 24 hrs-MII oocytes were analysed by dUDP nick-end labelling (TUNEL) markers and counterstained with DAPI. The embryos showed a varying degree of DNA fragmentation. While blastocyst stage embryos in general had 1 or 2 cells with positive TUNEL results in all groups, the majority of blocked embryos (Figure 3 and Figure 4) had fragmented and scattered DNA, which was rarely associated with a positive TUNEL outcome. The frequency of cell fragmentation without DNA was evident in embryos derived from IVM-MII or 13 hrs-MII and 24 hr-MII oocytes. However, in blocked embryos derived from IVM-MII oocytes, most of the chromatin was found intact. Blastocyst stage embryos from IVM-MII, OV-MII and 13 hrs-MII had 1–2 cells with positive TUNEL labelling (Figure 5). Although no cell number was examined, blastocysts derived from IVM-MII seemed smaller in size and with fewer cells within them (Figure 5).

## Discussion

The present study successfully detailed differences in oocytes of different maturation and in-vitro age following ICSI and IVF in their ability to form male and female pronuclei, initiate embryo division cycles and development to blastocysts in-vitro. We also identified differences in oolemma stability related to maturation and aging. The link between the ability of the cytoplasm to remodel the sperm chromatin to form pronuclei, and the maturation stage of oocytes, is well accepted. Studies in humans and mice, including the present study, have shown that the cytoplasm with the least ability to induce sperm decondensation is the immature GV stage cytoplasm [3]. Sperm may penetrate GV, or maturing oocytes, but they do not undergo detectable decondensation and the plasma membrane remains intact. Sperm heads located within maturing metaphase I oocytes undergo partial decondensation, whereas, in mature MII oocytes they complete the decondensation process.

In the present study, the swelling of the sperm and formation of pronuclei observed in IVM-MII, OV-MII and 13 hrs-MII oocytes did not correlate with the developmental competence of these oocytes in-vitro. While step wise culture systems for in-vitro maturation of young oocytes has been suggested to be most optimal [18], simple solutions and mixture of simple solutions with more complex ones in a 1:1 ratio, where found more supportive of oocyte competence for IVM when oocytes retrieved after hormonal induction [19]. In the present study we deliberately used simple salt solution to eliminate corrections of in-vitro maturation and ageing by complex solutions.

The capacity of IVM-MII oocytes to progress to the blastocyst stage in-culture was more limited following ICSI. This may be related to the increased incidence of abnormal configuration of chromatin and meiotic spindles observed in IVM mouse oocytes [20], or to the compromised capacity of IVM-MII oocytes to be activated and initiate normal embryonic cell cycles, more so by ICSI than by IVF. It is recognised that the oocyte is transcriptionally silenced during maturation [21] so the increasing capacity to remodel sperm chromatin must derive from post-translational activation of bioactive factors during maturation, which become exaggerated in their ability to form pronuclei or even pseudo-pronuclei with ageing. In mice, the absolute rate of protein synthesis decreases during maturation in-vitro [22]. Injection of sperm directly into the cytoplasm bypasses the oocyte activation mechanisms that takes place following sperm-oocyte fusion [23]. Although this has no major effect on embryo development when ovulated oocytes are used, the impact on IVM-MII oocytes may be crucial. Similar observations have been made in humans where fertilization rates increased following IVM and ICSI but embryo quality, implantation and pregnancy rates were significantly reduced, when compared to IVF of IVM oocytes [24,25]. Similarly in the present study, fewer blastocysts were obtained from ICSI of IVM-MII oocytes, therefore limiting the number of embryos that can be transferred and the resultant number of live offspring. Calcium plays a role in the regulation of diverse cellular functions including enhancement of oocyte maturation and embryo development [26]. Dysfunction in calcium release and intracellular mobilization may affect embryo developmental competence [27]. Not only IVM-MII oocytes have reduced capacity to incorporate, release and use calcium efficiently [28], they also have reduced mitochondrial polarization [18]. Abnormal function of the mitochondria may lead to suboptimal ATP resulting in developmental incompetence related to energy deficiency as clearly evident in IVM-MII oocytes.

Oocytes found to be more resilient to injury with maturation and in-vitro ageing. The well documented phenomenon of zona hardening [29,30] is not related to oocyte maturation stage but to the length of oocyte culture in-vitro. Here we observed oolemma stability with maturation and ageing. The capacity to reseal the plasma membrane is associated with the intracellular membrane (endomembrane) and is a calcium dependent mechanism [31]. Calcium is required in the external environment for the resealing of the plasma membrane after ICSI. In GV and some IVM oocytes, the calcium channels responsible for calcium import from external sources (voltage-dependent, N-type and L-type) are not uniformly distributed in the membrane when compared to freshly ovulated oocytes [32]. The distribution of these channels is rearranged during maturation from localised fractions to a uniformed distribution. These channels are active at the early cleavage stages of embryo development up until the blastocyst stage where they disappear [32]. An inability to efficiently import calcium to the site of injury will result in cell death. This was evident in GV oocytes. Nonetheless, resistance to injury is not related to the capacity of the oocyte to complete development to blastocysts. Aged oocytes which displayed high survival rate after ICSI did not necessarily developed to blastocysts at high rates.

Embryos from 24 hrs-MII oocytes are either parthenogenones or polyspermic. In this group, formation of female pronuclei in most oocytes occurred before sperm nuclei underwent decondensation and there was no second polar body extrusion. It is also known that aged oocytes are less capable of blocking polyspermy [33]. Hence, abnormal development may also be due to polyspermia following IVF. Other abnormalities associated with aged oocytes are related to calcium metabolism. In in-vivo aged mouse oocytes obtained from the oviducts at 18.5 hr after hCG injection the frequency of calcium oscillations following fertilization significantly increases compare to freshly ovulated (12.5 hr post hCG injection) mouse oocytes [10,13]. However, the amplitudes of the oscillations are smaller than in freshly ovulated oocytes. Aged oocytes are unable to appropriately regulate ATP levels following fertilization as a result of exposure of the postovulatory oocytes to prolonged oxidative stresses within the oviducts or prolonged in-vitro cultures as in the present study [34]. The calcium signal following fertilization and activation of mouse 24 hrs aged oocytes results in the activation of caspase, a sign of apoptosis and coincidently, the antiapoptotic Bcl-2 protein is reduced. In our study we identified empty cell fragments evident in embryos derived from IVM-MII and excessively aged oocytes (24 hrs-MII). This may be due to the significant differences in spindle position and distribution in IVM and aged 24 hrs oocytes when compared to freshly ovulated oocytes [35,36]. As a consequence this results, in high proportion of aneuploidy [37]. Similar to the observations by Van Blerkom and Davis [38], who examined apoptosis in aged unfertilised oocytes up to 5 days, DNA fragmentation within cellular fragments of embryos that resulted from 24 hrs aged oocytes or blocked/fragmented embryos from all the groups were rarely associated with positive dUDP nick-end labelling (TUNEL) marker. Van Blerkom and Davis [38] identified TUNEL positive fragments under the zona pellucida, which were likely to be related to fragmentation of the first polar body. It may be that identifying caspase activity [10,39] within blocked and fragmented mouse embryos is a more accurate way to predict programmed cell death.

Oocytes that were aged for 13 hrs only did not reach the blastocyst stage at similar rates to OV-MII oocytes. These oocytes are part-way through the aging process and they start losing control over calcium and other metabolic pathways. Low fertilization and pregnancy rates using human oocytes reinseminated by ICSI, 25 hrs after oocyte collection has also been reported in the past [40-42]. When human oocytes are rescued 19–22 hrs after retrieval [43], fertilization, embryo development and pregnancy rates are relatively higher than oocytes rescued at 24–30 hrs after retrieval [40-42]. Even better outcome is established when human oocytes are rescued 9 hrs from egg retrieval [44].

In addition to the ageing period, culture media, storage temperature and atmosphere have a strong relationship to developmental competence of aged oocytes [11]. Keeping 24 hrs old mouse oocytes in darkness at room temperature in simple solution such as modified Kreb-Ringer bicarbonate known as TYH medium, allows a limited ability to retain full developmental potential [11]. Although simple solution was used in the present study to age oocytes for 24 hrs, they were kept in 37°C. Even if their capacity to progress in embryonic development will be maintained, few reports indicate that post ovulatory ageing is associated with abnormal gene expression and epigenetic alternations [45,46].

## Conclusions

Physiological differences are evident between oocytes at different maturation and ageing stages. These, no doubtfully are associated with biological processes taking place during maturation and ageing. More importantly is the clear acknowledgement of the superiority of freshly ovulated MII mouse oocytes over IVM-MII or aged MII oocytes, in relation to developmental competence. Attempts to advance development by culture conditions that may improve maturation and slow aging in-vitro, results in only a small enhancement of embryo development in the mouse [10]. This may raise concerns when IVM oocytes and aged (mainly failed fertilized) oocytes are used in human IVF or for nuclear transfer to form embryonic stem cells [44-47].

## Competing interests

The authors declare that they have no competing interests.

## Authors' contributions

OLK designed and preformed the experiments, provided intellectual input, and participated in writing the manuscript. AT provided intellectual input and participated in writing the manuscript.
